# Supplementary material for: Playing it safe: information constrains collective betting strategies
Source: ArXiv. 2023 May 28:arXiv:2304.08976v2. Originally published 2023 Apr 18. Preprint. [Version 2] (PMC10153290)
Supplement: Supplement 1 [file NIHPP2304.08976v2-supplement-1.pdf]

Appendix A: Jeffreys prior for discrete and continuous distributions

We compute Jeffreys prior for common examples of discrete and continuous distributions. To this end, we define the Fisher information metric (FIM) as the coefficient of the second order term in the Taylor expansion of the Kullback-Leibler (KL) divergence Eq. (1). If two parameterised distributions, are defined by discrete sets of parameters $\boldsymbol{\theta} = \{\theta_1 \ldots, \theta_d\}$ and $\boldsymbol{\phi} = \{\phi_1 \ldots, \phi_d\}$, respectively, we consider the Taylor expansion of the KL divergence between two models which are close in parameter space $\boldsymbol{\phi} \equiv \boldsymbol{\theta} - \delta\boldsymbol{\theta}$:

$$D_{\mathrm{KL}}(P_{\boldsymbol{\theta}} || P_{\boldsymbol{\theta}-\delta\boldsymbol{\theta}}) = \frac{1}{2}\mathcal{I}_{ij}\delta\theta^i\delta\theta^j + \ldots \,, \tag{A1}$$

where summation over repeated indices is implied and $\mathcal{I}$ is the FIM, given by

$$\mathcal{I}_{ij}(\boldsymbol{\theta}) = \partial_{\delta\theta^i}\partial_{\delta\theta^j} D_{\mathrm{KL}}(P_{\boldsymbol{\theta}} || P_{\boldsymbol{\theta}-\delta\boldsymbol{\theta}})\Big|_{\delta\boldsymbol{\theta}=\mathbf{0}} \,. \tag{A2}$$

The normalised Jeffreys prior is computed from the FIM through the relation

$$w(\boldsymbol{\theta}) = \frac{\sqrt{\det \mathcal{I}(\boldsymbol{\theta})}}{\int \mathrm{d}^d\boldsymbol{\psi} \sqrt{\det \mathcal{I}(\boldsymbol{\psi})}} \,. \tag{A3}$$

1. Multinomial distribution

We define the multinomial distributions with parameters $\boldsymbol{\theta}$ and $\boldsymbol{\theta}'$ as:

$$P_{\boldsymbol{\theta}}(\mathbf{x}) = \frac{1}{B(\mathbf{x})}\prod_{i=1}^{d}\theta_i^{x_i} \,,\ Q_{\boldsymbol{\theta}'}(\mathbf{x}) = \frac{1}{B(\mathbf{x})}\prod_{i=1}^{d}{\theta'}_i^{x_i} \tag{A4}$$

with

$$\sum_{i=1}^{d} \theta_i = \sum_{i=1}^{d} {\theta'}_i = 1\,,\ x_i \in \{0, ..., N\} \text{ and } \sum_{i=1}^{d} x_i = N\,. \tag{A5}$$

The KL divergence between the two distributions is given by

$$D_{\mathrm{KL}}(P_{\boldsymbol{\theta}}||Q_{\boldsymbol{\theta}'}) = \sum_{\mathbf{x}} \frac{1}{B(\mathbf{x})} \prod_{i=1}^{d} \theta_i^{x_i} \log\left(\frac{\prod_j \theta_j^{x_j}}{\prod_j {\theta'}_j^{x_j}}\right) \tag{A6}$$

$$= \sum_j (\log\theta_j - \log\theta'_j)\left(\sum_{\mathbf{x}} \frac{x_j}{B(\mathbf{x})} \prod_{i=1}^{d} \theta_i^{x_i}\right) \tag{A7}$$

$$= \sum_j (\log\theta_j - \log\theta'_j) N\theta_j\,, \tag{A8}$$

where in the last step we have evaluated the mean of the multinomial distribution. The next step is to Taylor expand the KL divergence around $\boldsymbol{\theta}$, by setting $\boldsymbol{\theta}' = \boldsymbol{\theta} - \delta\boldsymbol{\theta}$. For the Taylor expansion we find

$$D_{\mathrm{KL}}(P_{\boldsymbol{\theta}}||Q_{\boldsymbol{\theta}'}) = \sum_j \left(\frac{\delta\theta_j}{\theta_j} + \frac{\delta\theta_j^2}{2\theta_j^2} + O(\delta\theta_j^3)\right) N\theta_j = \sum_j \left(\frac{\delta\theta_j^2}{2\theta_j^2} + O(\delta\theta_j^3)\right) N\theta_j\,, \tag{A9}$$

where in the last step the linear term vanishes since $\sum_i \theta_i = \sum_i \theta'_i = 1$ and $\theta'_i = \theta_i - \delta\theta_i$. From the quadratic term we read off the form of the FIM

$$\mathcal{I}_{ij}(\boldsymbol{\theta}) = N\frac{\delta_{ij}}{\theta_i}\,, \tag{A10}$$

where δ_{ij} is the Kronecker delta. Taking the square root of the determinant and computing the normalisation factor finally gives us Jeffreys prior:

$$w(\boldsymbol{\theta}) = Dir(\boldsymbol{\theta}; 1/2, \ldots, 1/2) = \frac{\Gamma(d/2)}{\sqrt{\pi}^d} \frac{1}{\sqrt{1 - \sum_{i=1}^{d-1}\theta_i}} \prod_{i=1}^{d-1} \frac{1}{\sqrt{\theta_i}}\,, \tag{A11}$$

where Dir is the Dirichlet distribution.

2. Poisson distribution

We consider two Poisson distributions with parameters λ' and λ:

$$P_{\lambda'}(n) = \frac{{\lambda'}^n e^{-\lambda'}}{n!}\,,\ Q_\lambda(n) = \frac{\lambda^n e^{-\lambda}}{n!}\,. \tag{A12}$$

The KL divergence between the two distributions is given by

$$D_{\mathrm{KL}}(P_{\lambda'}||Q_\lambda) = \sum_n \frac{{\lambda'}^n e^{-\lambda'}}{n!} \log\left(\frac{{\lambda'}^n e^{-\lambda'}}{n!}\frac{n!}{\lambda^n e^{-\lambda}}\right) = \lambda' \log\frac{\lambda'}{\lambda} - \lambda' + \lambda\,. \tag{A13}$$

The next step is to Taylor expand the KL divergence around λ', by setting $\lambda = \lambda' + \delta\lambda$. For the Taylor expansion we find

$$D_{\mathrm{KL}}(P_{\lambda'}||Q_\lambda) = \frac{\delta\lambda^2}{2\lambda'} + O(\delta\lambda^3)\,. \tag{A14}$$

From the quadratic term we read off the form of the FIM

$$\mathcal{I}(\lambda') = \frac{1}{\lambda'}\,. \tag{A15}$$

Taking the square root gives us Jeffreys prior:

$$w(\lambda') = \frac{1}{\sqrt{\lambda'}}\,, \tag{A16}$$

Jeffreys prior is shown in Figure S1. It diverges on the boundary of parameter space for $\lambda' \to 0$ and approach zero as for $\lambda' \to \infty$.

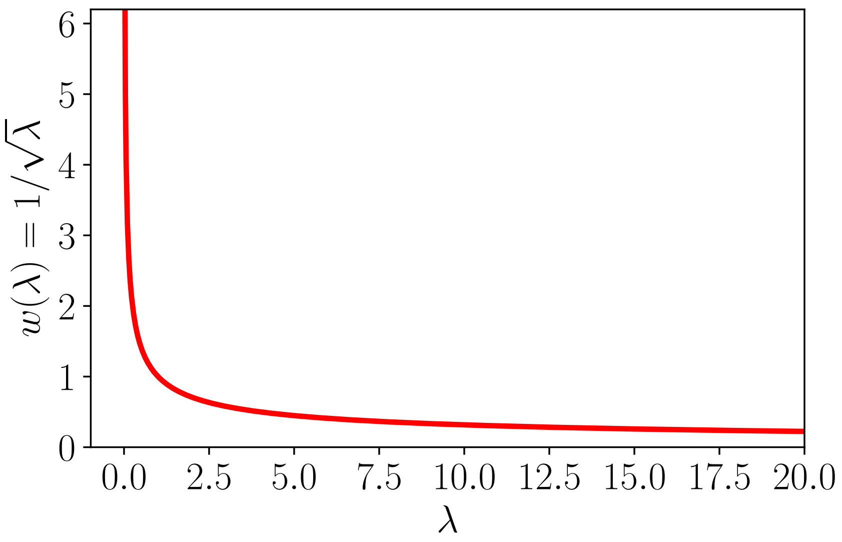

FIG. S1. Jeffreys prior for the Poisson distribution with rate λ, diverges at the boundary of parameter space for $\lambda \to 0$.

3. Gauss distribution

The KL divergence for continuous distributions is defined as

$$D_{\rm KL}(P||Q) = \int \mathrm{d}x\, P(x) \log \frac{P(x)}{Q(x)}\,. \tag{A17}$$

The KL divergence between two Gaussian distributions with mean μ and variances σ^2 and σ'^2

$$P_{\mu,\sigma^2}(x) = \frac{1}{\sqrt{2\pi\sigma^2}} e^{-\frac{(x-\mu)^2}{2\sigma^2}}\,,\; P_{\mu,\sigma'^2}(x) = \frac{1}{\sqrt{2\pi\sigma'^2}} e^{-\frac{(x-\mu)^2}{2\sigma'^2}}\,, \tag{A18}$$

is given by

$$D_{\rm KL}(P_{\mu,\sigma^2}||P_{\mu,\sigma'^2}) = -\frac{1}{2}\left[\log\sigma^2 - \log\sigma'^2 + 1 - \frac{\sigma^2}{\sigma'^2}\right]\,. \tag{A19}$$

The next step is to Taylor expand the KL divergence around σ, by setting $\sigma' = \sigma + \delta\sigma$. For the Taylor expansion we find

$$D_{\rm KL}(P_{\mu,\sigma^2}||P_{\mu,\sigma'^2}) = \frac{1}{2}\left[\partial^2_{\sigma'} D_{\rm KL}(P_{\mu,\sigma^2}||P_{\mu,\sigma'^2})\right]_{\delta\sigma=0}\delta\sigma^2 + O(\delta\sigma^3) = -\frac{1}{4}\left[\frac{2}{\sigma'^2} - 6\frac{\sigma^2}{\sigma'^4}\right]_{\delta\sigma=0}\delta\sigma^2 + O(\delta\sigma^3) \tag{A20}$$

From this we find the FIM:

$$\mathcal{I}(\sigma) = \frac{1}{\sigma^2} \tag{A21}$$

and Jeffreys prior

$$w(\sigma) = \frac{1}{\sigma}\,. \tag{A22}$$

Jeffreys prior for the Gaussian is shown in Figure S2. It diverges on the boundary of parameter space as $\sigma \to 0$ and approaches zero for $\sigma \to \infty$.

Appendix B: Standard error and diverging KL divergence

Consider the KL divergence for the Bernoulli model

$$D_{\rm KL}(P_{\theta^*}||P_\theta) = H_{\theta^*}(\theta) - H_{\theta^*}(\theta^*)\,, \tag{B1}$$

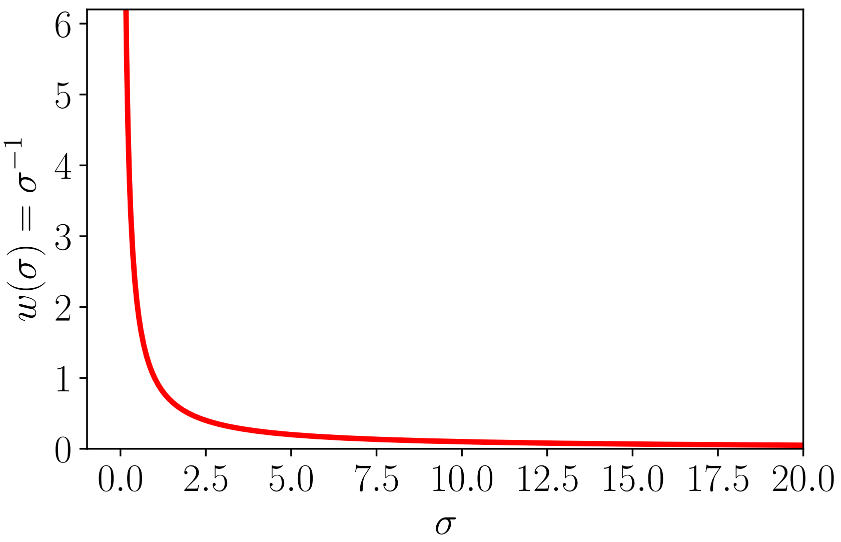

FIG. S2. Jeffreys prior for the Gaussian model with variance σ^2, diverges at the boundary of parameter space for $\sigma \to 0$.

where $H_x(y)$ is the cross-entropy function

$$H_x(y) = -x \log y - (1-x)\log(1-y)\,. \tag{B2}$$

For $\theta \to 0, 1$, the KL divergence diverges to plus infinity. What is the behaviour of the KL divergence when we try to estimate the value of θ using data from many Bernoulli trials generated by the true model θ^*? In particular, how does the KL divergence behave when θ^* lies very close to a boundary of parameter space?

To answer this question we consider the empirically estimated parameter $\hat{\theta}$. This parameter is subject to statistical fluctuations due to finite data size. In general, the standard error for a parameter estimated from N independent trials is given by

$$\sigma_N = \frac{\sigma}{\sqrt{N}}\,, \tag{B3}$$

where σ is the standard deviation of the distribution we are sampling from. Within the standard error the estimated parameter lies within the bounds

$$\hat{\theta}_\pm = \theta^* \pm \sigma_N\,. \tag{B4}$$

For the Bernoulli model, the empirical estimate of the probability parameter is obtained as the mean number of events observed in N independent Bernoulli trials and σ is the standard deviation of the Bernoulli distribution

$$\sigma = \sqrt{\theta^*(1-\theta^*)} = \theta^* \sqrt{1/\theta^* - 1}\,. \tag{B5}$$

The statistically expected bounds are

$$\hat{\theta}_\pm = \theta^* \left(1 - \sqrt{\frac{1/\theta^* - 1}{N}}\right)\,. \tag{B6}$$

Let us now focus on the boundary at $\theta = 0$ and recall that the KL divergence diverges at this boundary. Therefore, one way to check whether we can expect the KL divergence to diverge for a given model θ^* and number of observations N, is to check whether the boundary $\theta = 0$ lies within the standard error bound $\hat{\theta}_-$. From the last equation we see that the boundary is included if the condition

$$\sqrt{\frac{1/\theta^* - 1}{N}} > 1 \tag{B7}$$

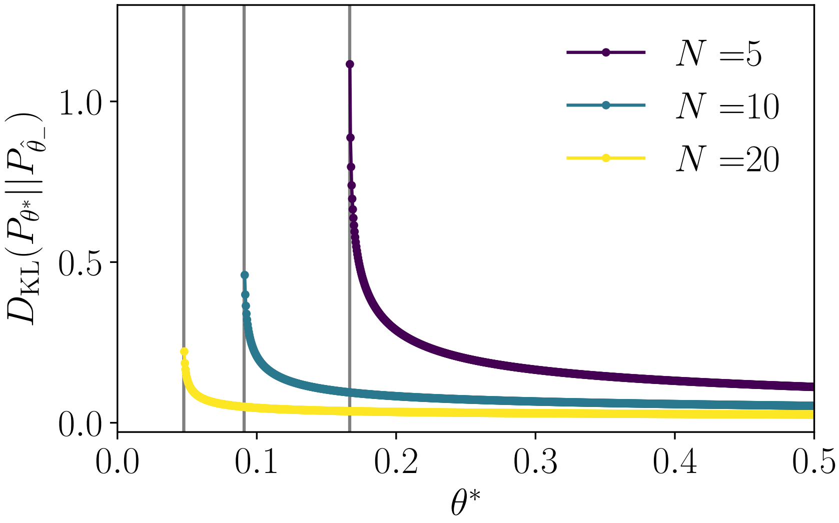

FIG. S3. Diverging behaviour of $D_{\mathrm{KL}}(P_{\theta^*}||P_{\hat{\theta}_-=\theta^*-\sigma_N})$ for three values of N. Vertical gray lines show the position of diverging behaviour at $1/(N+1)$.

is satisfied. Rearranging this inequality gives

$$\theta^* < \frac{1}{N+1}\,. \tag{B8}$$

Thus true models which are within $1/(N+1)$ of the boundary, the boundary where the KL divergence diverges lies within the standard error and statistically we can expect the KL divergence to diverge. See Figure S3 for examples plots.

For the Poisson model, the empirical estimate of the parameter lambda is obtained as the mean number of events observed in N independent Poisson trials and σ is the standard deviation of the Poisson distribution

$$\sigma_N = \sqrt{\frac{\lambda^*}{N}} = \lambda^* \frac{1}{\sqrt{\lambda^* N}}\,. \tag{B9}$$

The statistically expected bounds are

$$\hat{\lambda}_{\pm} = \lambda^* \pm \sigma_N = \lambda^* \left(1 \pm \frac{1}{\sqrt{\lambda^* N}}\right)\,. \tag{B10}$$

The boundary of parameter space lies at $\lambda = 0$. Therefore we consider $\hat{\lambda}_-$ as the important bound and the condition for the boundary of parameter space to be included is

$$\frac{1}{\sqrt{\lambda^* N}} > 1\,, \tag{B11}$$

or

$$\lambda^* < \frac{1}{N}\,. \tag{B12}$$

Therefore, for any given N we can find a models λ^* which lie close enough to the boundary according to the above condition, and for which we should expect the KL divergence to diverge.

Appendix C: Optimality condition

1. The optimality condition for exponential families in mean parameterisation

This derivation makes use of the fact that the Kullback-Leibler divergence between two distributions from the same exponential family in canonical form, can be expressed as the Bregman divergence (defined with respect to the dual

of the log partition function, F^*) between the mean parameters $\boldsymbol{\theta}$:

$$D_{\mathrm{KL}}(Q_{\boldsymbol{\eta}^*}||Q_{\boldsymbol{\eta}}) = B_{F^*}(\boldsymbol{\theta}^* : \boldsymbol{\theta})\,. \tag{C1}$$

Writing out the definition of the Bregman divergence, the right-hand side is of the form

$$B_{F^*}(\boldsymbol{\theta}^* : \boldsymbol{\theta}) = F^*(\boldsymbol{\theta}^*) - F^*(\boldsymbol{\theta}) - [\boldsymbol{\theta}^* - \boldsymbol{\theta}]^T \cdot \nabla_{\boldsymbol{\theta}} F^*(\boldsymbol{\theta})\,, \tag{C2}$$

where

$$\boldsymbol{\theta} = \kappa \boldsymbol{\theta}_{\mathrm{bias}} + (1-\kappa)\hat{\boldsymbol{\theta}}_{\mathrm{ML}}\,. \tag{C3}$$

To compute the condition for the optimal model, we take the derivative of the Bregman divergence with respect to the bias:

$$\frac{1}{\kappa}\nabla_{\theta_{\mathrm{bias}\,i}} B_{F^*}(\boldsymbol{\theta}^* : \boldsymbol{\theta}) = \nabla_{\theta_i} B_{F^*}(\boldsymbol{\theta}^* : \boldsymbol{\theta}) = -\nabla_{\theta_i} F^*(\boldsymbol{\theta}) - \boldsymbol{\theta}^{*T} \cdot \nabla_{\theta_i}\nabla_{\boldsymbol{\theta}} F^*(\boldsymbol{\theta}) + \nabla_{\theta_i}\{\boldsymbol{\theta}^T \cdot \nabla_{\boldsymbol{\theta}} F^*(\boldsymbol{\theta})\} \tag{C4}$$

$$= -\nabla_{\theta_i} F^*(\boldsymbol{\theta}) - \sum_{j=1}^{d} \theta_j^* \nabla_{\theta_i}\nabla_{\theta_j} F^*(\boldsymbol{\theta}) + \nabla_{\theta_i} F^*(\boldsymbol{\theta}) + \sum_{j=1}^{d} \theta_j \nabla_{\theta_i}\nabla_{\theta_j} F^*(\boldsymbol{\theta}) \tag{C5}$$

$$= -\sum_{j=1}^{d} [\theta_j^* - \theta_j] \nabla_{\theta_i}\nabla_{\theta_j} F^*(\boldsymbol{\theta})\,. \tag{C6}$$

Next, we use the relation

$$\mathcal{I}_{ij}(\boldsymbol{\theta}) = \nabla_{\theta_i}\nabla_{\theta_j} F^*(\boldsymbol{\theta})\,, \tag{C7}$$

relating the Fisher information metric in mean parameterization to the dual function. We derive this relation starting with Eq. (8)

$$D_{\mathrm{KL}}(Q_{\boldsymbol{\eta}^*(\boldsymbol{\theta}^*)}||Q_{\boldsymbol{\eta}(\boldsymbol{\theta})}) = B_{F^*}(\boldsymbol{\theta}^* : \boldsymbol{\theta})\,, \tag{C8}$$

replacing $\boldsymbol{\theta} = \boldsymbol{\theta}^* - \delta\boldsymbol{\theta}$, and Taylor expanding in $\delta\boldsymbol{\theta}$. Using the definition of the Bregman divergence Eq. (C2) and noting that $\nabla_{\delta\theta_i} = -\nabla_{\theta_i}$, the coefficients of the first three orders in $\delta\boldsymbol{\theta}$ are given by

$$B_{F^*}(\boldsymbol{\theta}^* : \boldsymbol{\theta}^* - \delta\boldsymbol{\theta})\big|_{\delta\boldsymbol{\theta}=0} = 0\,, \tag{C9}$$

$$\nabla_{\delta\theta_i} B_{F^*}(\boldsymbol{\theta}^* : \boldsymbol{\theta}^* - \delta\boldsymbol{\theta})\big|_{\delta\boldsymbol{\theta}=0} = \left[\nabla_{\theta_i} F^*(\boldsymbol{\theta}) - \nabla_{\theta_i} F^*(\boldsymbol{\theta}) + \sum_j \delta\theta_j \nabla_{\theta_i}\nabla_{\theta)j} F^*(\boldsymbol{\theta})\right]_{\delta\boldsymbol{\theta}=0} = 0\,, \tag{C10}$$

$$\nabla_{\delta\theta_k}\nabla_{\delta\theta_i} B_{F^*}(\boldsymbol{\theta}^* : \boldsymbol{\theta}^* - \delta\boldsymbol{\theta})\big|_{\delta\boldsymbol{\theta}=0} = \Big[- \nabla_{\delta\theta_k}\nabla_{\theta_i} F^*(\boldsymbol{\theta}) + \nabla_{\delta\theta_k}\nabla_{\theta_i} F^*(\boldsymbol{\theta}) + \delta_{kj}\nabla_{\theta_i}\nabla_{\theta_j} F^*(\boldsymbol{\theta}) \tag{C11}$$

$$- \sum_j \delta\theta_j \nabla_{\delta\theta_k}\nabla_{\theta_i}\nabla_{\theta_j} F^*(\boldsymbol{\theta})\Big]_{\delta\boldsymbol{\theta}=0} = \nabla_{\theta_i}\nabla_{\theta_k} F^*(\boldsymbol{\theta}^*)\,. \tag{C12}$$

Using this relation, we rewrite the above condition as

$$\frac{1}{\kappa}\nabla_{\boldsymbol{\theta}_{\mathrm{bias}}} B_{F^*}(\boldsymbol{\theta}^* : \boldsymbol{\theta}) = -\mathcal{I}(\boldsymbol{\theta})\,[\boldsymbol{\theta}^* - \boldsymbol{\theta}] = \mathcal{I}(\boldsymbol{\theta})\delta\boldsymbol{\theta}\,, \tag{C13}$$

where $-\delta\boldsymbol{\theta} \equiv \boldsymbol{\theta}^* - \boldsymbol{\theta}$. Thus, in mean parameterisation, the optimality constraint in Eq. (6) of the main text, takes the simple form

$$\frac{1}{\kappa}\nabla_{\boldsymbol{\theta}_{\mathrm{bias}}} \big\langle \mathcal{L}_{\boldsymbol{\theta}^*}(\boldsymbol{\theta}) \big\rangle_{\hat{\boldsymbol{\theta}}_{\mathrm{ML}}} = \big\langle \mathcal{I}(\boldsymbol{\theta})\delta\boldsymbol{\theta} \big\rangle_{\hat{\boldsymbol{\theta}}_{\mathrm{ML}}} = 0\,. \tag{C14}$$

2. The optimality condition for the Bernoulli model in mean parameterization derived from Taylor expansion

As an example, we evaluate the optimality condition

$$\nabla_{\boldsymbol{\theta}_{\mathrm{bias}}} \big\langle D_{\mathrm{KL}}(P_{\boldsymbol{\theta}^*}||P_{\boldsymbol{\theta}}) \big\rangle_{\hat{\boldsymbol{\theta}}_{\mathrm{ML}}} = 0 \tag{C15}$$

for the Bernoulli model written in mean parameters. In this derivation we will use Taylor expansion of the KL divergence and find that all but a single term of the infinite series cancel to yield a simplified form of the condition in Eq. (9) of main text.

We consider KL divergence between two Bernoulli distributions, denoted by $P_{\boldsymbol{\theta}^*}$ and $P_{\boldsymbol{\theta}}$, respectively:

$$D_{\mathrm{KL}}(P_{\boldsymbol{\theta}^*}||P_{\boldsymbol{\theta}}) = D_{\mathrm{KL}}(P_{\boldsymbol{\theta}+\delta\boldsymbol{\theta}}||P_{\boldsymbol{\theta}}) = \sum_x P_{\boldsymbol{\theta}+\delta\boldsymbol{\theta}} \log\left(\frac{P_{\boldsymbol{\theta}+\delta\boldsymbol{\theta}}}{P_{\boldsymbol{\theta}}}\right), \tag{C16}$$

where $\boldsymbol{\theta}^* \equiv \boldsymbol{\theta} + \delta\boldsymbol{\theta}$ and expand the right-hand side in $\delta\boldsymbol{\theta}$. For the first few orders we find

$$D_{\mathrm{KL}}(P_{\boldsymbol{\theta}^*}||P_{\boldsymbol{\theta}})\Big|_{\delta\boldsymbol{\theta}=0} = 0\,, \tag{C17}$$

$$\partial D_{\mathrm{KL}}(P_{\boldsymbol{\theta}^*}||P_{\boldsymbol{\theta}})\Big|_{\delta\boldsymbol{\theta}=0} = 1\,, \tag{C18}$$

$$\partial^2 D_{\mathrm{KL}}(P_{\boldsymbol{\theta}^*}||P_{\boldsymbol{\theta}})\Big|_{\delta\boldsymbol{\theta}=0} = 1/\boldsymbol{\theta}\,, \tag{C19}$$

$$\partial^3 D_{\mathrm{KL}}(P_{\boldsymbol{\theta}^*}||P_{\boldsymbol{\theta}})\Big|_{\delta\boldsymbol{\theta}=0} = -1/\boldsymbol{\theta}^2\,, \tag{C20}$$

$$\partial^4 D_{\mathrm{KL}}(P_{\boldsymbol{\theta}^*}||P_{\boldsymbol{\theta}})\Big|_{\delta\boldsymbol{\theta}=0} = 2/\boldsymbol{\theta}^3\,, \tag{C21}$$

$$\partial^5 D_{\mathrm{KL}}(P_{\boldsymbol{\theta}^*}||P_{\boldsymbol{\theta}})\Big|_{\delta\boldsymbol{\theta}=0} = -6/\boldsymbol{\theta}^4\,, \tag{C22}$$

$$\vdots$$

where $\partial \equiv \partial_{\delta\boldsymbol{\theta}}$ and powers on the right-hand side act element-wise. The Taylor expansion is thus

$$D_{\mathrm{KL}}(P_{\boldsymbol{\theta}^*}||P_{\boldsymbol{\theta}}) = \sum_i \left\{\delta\theta_i + \frac{1}{2!}\frac{\delta\theta_i^2}{\theta_i} - \frac{1}{3!}\frac{\delta\theta_i^3}{\theta_i^2} + \frac{1}{4!}\frac{2\delta\theta_i^4}{\theta_i^3} - \frac{1}{5!}\frac{6\delta\theta_i^5}{\theta_i^4} + O(\delta_i^6)\right\} \tag{C23}$$

$$= 1 + \sum_i \left\{\frac{1}{2!}\frac{\delta\theta_i^2}{\theta_i} - \frac{1}{3!}\frac{\delta\theta_i^3}{\theta_i^2} + \frac{1}{4!}\frac{2\delta\theta_i^4}{\theta_i^3} - \frac{1}{5!}\frac{6\delta\theta_i^5}{\theta_i^4} + O(\delta_i^6)\right\} \tag{C24}$$

Next, we act with the derivative with respect to the bias:

$$\partial_{\theta_{\mathrm{bias}\,i}} D_{\mathrm{KL}}(P_{\boldsymbol{\theta}^*}||P_{\boldsymbol{\theta}}) = (\partial\theta_i/\partial\theta_{\mathrm{bias}\,i})\partial_{\theta_i} D_{\mathrm{KL}}(P_{\boldsymbol{\theta}^*}||P_{\boldsymbol{\theta}}) = \kappa\partial_{\theta_i} D_{\mathrm{KL}}(P_{\boldsymbol{\theta}^*}||P_{\boldsymbol{\theta}})\,. \tag{C25}$$

Remembering that $\delta\boldsymbol{\theta}$ is a function of $\boldsymbol{\theta}$, $\partial\delta\theta/\partial\theta = -1$, we obtain:

$$\partial_{\theta_i} D_{\mathrm{KL}}(P_{\boldsymbol{\theta}^*}||P_{\boldsymbol{\theta}}) = -\frac{1}{\theta_i}\delta\theta_i + \left[-\frac{1}{2!}\frac{\delta\theta_i^2}{\theta_i^2} + \frac{3}{3!}\frac{\delta\theta_i^2}{\theta_i^2}\right] + \left[\frac{2}{3!}\frac{\delta\theta_i^3}{\theta_i^3} - \frac{4}{4!}\frac{2\delta\theta_i^3}{\theta_i^3}\right] + \ldots \tag{C26}$$

$$= -\frac{1}{\theta_i}\delta\theta_i\,, \tag{C27}$$

where on in the first line on the right-hand side we have grouped terms such that expressions in the brackets vanish to show how terms of successive order in $\delta\theta$ cancel. Using $\theta_2 = 1 - \theta_1$, we finally see that in matrix notation the expression takes the form

$$\partial_{\boldsymbol{\theta}} D_{\mathrm{KL}}(P_{\boldsymbol{\theta}^*}||P_{\boldsymbol{\theta}}) = -\begin{pmatrix} 1/\theta_1 & 0 \\ 0 & 1/(1-\theta_1) \end{pmatrix} \delta\boldsymbol{\theta} = -\mathcal{I}(\boldsymbol{\theta})\delta\boldsymbol{\theta}\,, \tag{C28}$$

where we recognised the 2×2 matrix to be the Fisher information matrix. Thus, we obtain the optimality condition

$$-\big\langle\partial_{\boldsymbol{\theta}} D_{\mathrm{KL}}(P_{\boldsymbol{\theta}^*}||P_{\boldsymbol{\theta}})\big\rangle_{\hat{\boldsymbol{\theta}}_{\mathrm{ML}}} = \big\langle\mathcal{I}(\boldsymbol{\theta})\delta\boldsymbol{\theta}\big\rangle_{\hat{\boldsymbol{\theta}}_{\mathrm{ML}}} = 0\,. \tag{C29}$$

Appendix D: Optimal parameter calculation for Poisson distribution with unknown rate parameter

We consider two Poisson distributions with parameters λ^* and λ:

$$P_{\lambda^*}(n) = \frac{\lambda^{*\,n} e^{-\lambda^*}}{n!}\,,\ P_{\lambda}(n) = \frac{\lambda^n e^{-\lambda}}{n!}\,. \tag{D1}$$

λ_{bias}-optimization:

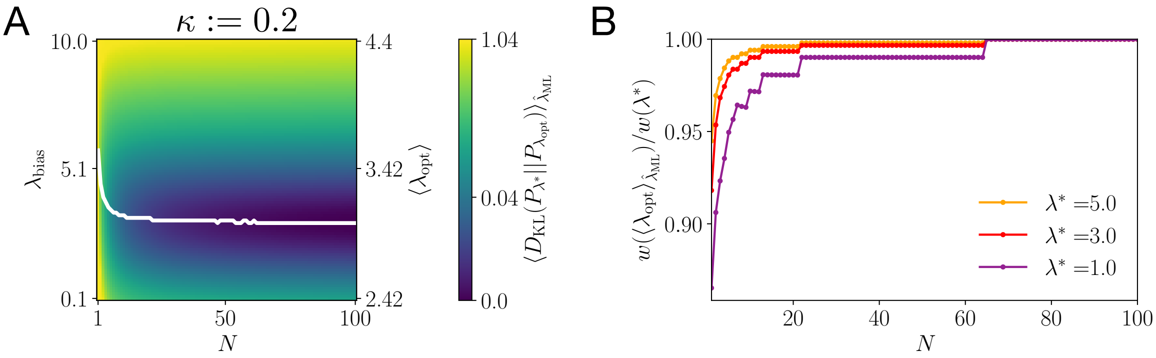

FIG. S4. Optimization of the expected loss for the Poisson distribution with respect to the prior shows a bias to low complexity models. The true model lies at $\lambda^* = 3$. (A) The bias λ_{bias} is optimised while $\kappa = 0.2$ ($q = 0.25$) is kept fixed. The plot shows the landscape of the expected loss as a function of the bias and the number of observations. We are using a non-linear colormap based on the cumulative distribution of the loss. (B) The plot shows for different true models λ^* the ratio of the local complexity measure by Jeffreys prior of the expected optimal to the true model.

The KL divergence between the two distributions is given by

$$D_{\text{KL}}(P_{\lambda^*}||Q_\lambda) = \sum_{n=0}^{\infty} \frac{{\lambda^*}^n e^{-\lambda^*}}{n!} \log\left(\frac{{\lambda^*}^n e^{-\lambda^*}}{n!}\frac{n!}{\lambda^n e^{-\lambda}}\right) = \lambda^* \log\frac{\lambda^*}{\lambda} + (\lambda - \lambda^*)\,. \tag{D2}$$

We want to minimise the posterior mean loss

$$\left\langle \mathcal{L}_{\lambda^*}\right\rangle_{\text{post}}(\lambda) = \int \mathrm{d}\tilde{\lambda} P(\tilde{\lambda}|\mathbf{n}) D_{\text{KL}}(P_{\tilde{\lambda}}||Q_\lambda) \tag{D3}$$

with respect to λ. Taking the derivative with respect to λ we obtain the condition

$$\partial_\lambda \left\langle \mathcal{L}_{\lambda^*}\right\rangle_{\text{post}} = \partial_\lambda \int \mathrm{d}\tilde{\lambda} P(\tilde{\lambda}|\mathbf{n}) D_{\text{KL}}(P_{\tilde{\lambda}}||Q_\lambda) = \int \mathrm{d}\tilde{\lambda} P(\tilde{\lambda}|\mathbf{n}) \left(-\tilde{\lambda}/\lambda + 1\right) = 0\,. \tag{D4}$$

The condition is satisfied by

$$\lambda = \int \mathrm{d}\tilde{\lambda} P(\tilde{\lambda}|\mathbf{n}) \tilde{\lambda}\,. \tag{D5}$$

The conjugate prior for the mean number of counts λ is the Gamma distribution and the posterior takes the form

$$P(\lambda|\mathbf{n}) = \text{Gamma}(\alpha', \beta') = \frac{{\beta'}^{\alpha'}}{\Gamma(\alpha')} \lambda^{\alpha'-1} e^{-\beta'\lambda} \tag{D6}$$

with

$$\alpha' = \alpha + N\bar{X} = \alpha + N\frac{1}{N}\sum_{i=1}^{N} x_i\,, \tag{D7}$$

$$\beta' = \beta + N\,, \tag{D8}$$

and the hyperparameters $\alpha, \beta > 0$. $\bar{X}$ is an empirical estimate of the rate λ from N observations. The posterior mean which gives the optimal parameter choice is given by

$$\lambda(\hat{\lambda}_{\text{ML}}; \alpha, \beta) = \frac{\alpha'}{\beta'} = \frac{\alpha + N\hat{\lambda}_{\text{ML}}}{\beta + N} = \frac{\frac{\beta}{N}\frac{\alpha}{\beta} + \hat{\lambda}_{\text{ML}}}{\frac{\beta}{N} + 1} = \frac{q\lambda_{\text{bias}} + \hat{\lambda}_{\text{ML}}}{q + 1} \tag{D9}$$

and we define as before

$$N_0 \equiv \beta\,, \quad \lambda_{\rm bias} \equiv \frac{\alpha}{\beta}\,, \quad \text{and} \quad q \equiv \frac{\beta}{N}\,. \tag{D10}$$

The hyperparameter β can thus be interpreted as virtual observations and α as the sum of counts across β observations. If we do not trust in the empirical estimate, then we should take α large compared to β. In this limit, the optimal λ values becomes large and lies further away from the boundary of parameter space situated at $\lambda = 0$.

Up to a scaling by $1/N$, the maximum-likelihood value of the average count $\hat{\lambda}_{\rm ML}$ computed from N observations, is distributed according to the true Poisson distribution with the rate rescaled by N. This can be seen from the characteristic function of the Poisson distribution given by $\exp(\lambda(e^{it}-1))$ and the fact that the characteristic function of the sum of N i.i.d. random variables is given by the N'th power of the characteristic function of a single random variable. The empirically averaged expected loss is then given by

$$\left\langle \mathcal{L}_{\lambda^*}(\lambda) \right\rangle_{\hat{\lambda}_{\rm ML}} = \sum_{n=0}^{\infty} {\rm Poi}(n; N\lambda^*) D_{\rm KL}(P_{\lambda^*} || Q_{\lambda(n/N;\alpha,\beta)})\,. \tag{D11}$$

We minimise this loss with respect to the hyperparameters, for instance by keeping β fixed an minimising with respect to α. In Figure S4 we show the optimal choice of the hyperparameter α as a function of the data size, confirming that for small N, α is large, while for increasing data size $\alpha \to 0$.

Appendix E: Optimal parameter calculation for Gaussian with known mean and unknown variance

Consider two Gaussian distributions with the same known mean μ and unknown variances ${\sigma^*}^2$ and σ^2

$$P_{{\sigma^*}^2}(x) = \frac{1}{\sqrt{2\pi{\sigma^*}^2}} e^{-\frac{(x-\mu)^2}{2{\sigma^*}^2}}\,,\; P_{\sigma^2}(x) = \frac{1}{\sqrt{2\pi\sigma^2}} e^{-\frac{(x-\mu)^2}{2\sigma^2}}\,. \tag{E1}$$

The KL divergence two between these distributions is given by

$$D_{\rm KL}(P_{{\sigma^*}^2} || P_{\sigma^2}) = -\frac{1}{2}\left[\log {\sigma^*}^2 - \log \sigma^2 + 1 - \frac{{\sigma^*}^2}{\sigma^2}\right]\,. \tag{E2}$$

We want to minimise the posterior mean loss

$$\left\langle \mathcal{L}_{\sigma^*} \right\rangle_{\rm post}(\sigma) = \int {\rm d}\tilde{\sigma}^2 P(\tilde{\sigma}^2|\mathbf{n}) D_{\rm KL}(P_{\tilde{\sigma}^2}||P_{\sigma^2}) \tag{E3}$$

with respect to σ^2, where $P(\tilde{\sigma}^2|\mathbf{n})$ is the posterior. Taking the derivative with respect to σ we obtain the condition

$$\partial_\sigma \int {\rm d}\tilde{\sigma}^2 P(\tilde{\sigma}^2|\mathbf{n}) D_{\rm KL}(P_{\tilde{\sigma}^2}||P_{\sigma^2}) = \int {\rm d}\tilde{\sigma}^2 P(\tilde{\sigma}^2|\mathbf{n}) \left(\frac{1}{\sigma} - \frac{\tilde{\sigma}^2}{\sigma^3}\right) = 0 \tag{E4}$$

satisfied by

$$\sigma^2 = \int {\rm d}\tilde{\sigma}^2 \tilde{\sigma}^2 P(\tilde{\sigma}^2|\mathbf{n})\,. \tag{E5}$$

The conjugate prior for the variance is the scaled inverse-chi-squared distribution χ^{-2}. In [56], the posterior distribution for the variance, is given as

$$P(\sigma^2|\nu_N, \sigma_N^2) = \chi^{-2}(\sigma^2|\nu_N, \sigma_N^2) = \frac{1}{\Gamma(\nu_N/2)}\left(\frac{\nu_N \sigma_N^2}{2}\right)^{\nu_N/2} x^{-\nu_N/2-1} e^{-\frac{\nu_N\sigma_N^2}{2x}}\,, \tag{E6}$$

where

$$\nu_N = \nu + N\,, \quad \text{with} \quad \nu > 2\,, \tag{E7}$$

$$\sigma_N^2 = \frac{1}{\nu_N}\left(\nu\sigma_0^2 + \sum_{i=1}^{N}(x_i-\mu)^2\right)\,. \tag{E8}$$

$\sigma^2_{\rm bias}$-optimization:

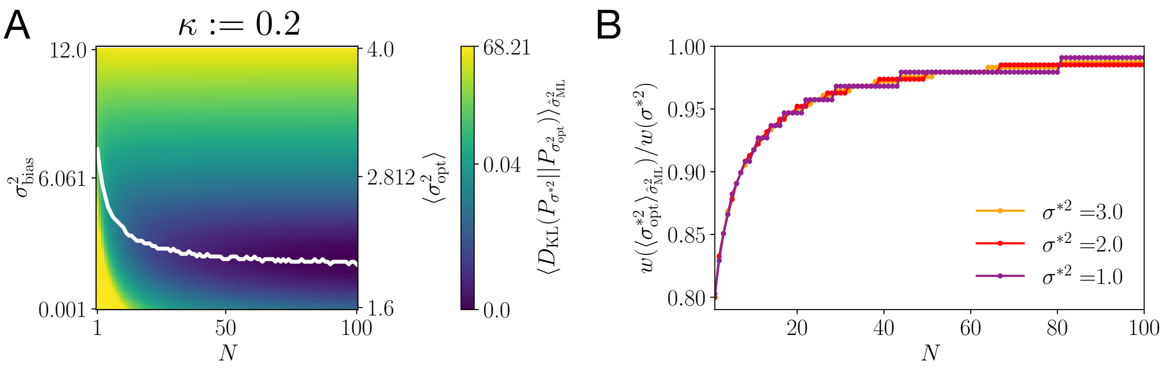

FIG. S5. Optimization of the expected loss for the Gaussian distribution with respect to the prior shows a bias to low complexity models. The true model lies at $\sigma^{*2} = 2$ and we set $\mu = 0$ for the known mean value. (A) The bias $\sigma^2_{\rm bias}$ is optimised while $\kappa = 0.2$ ($q = 0.25$) is kept fixed. The plot shows the landscape of the expected loss as a function of the bias and the number of observations. We are using a non-linear colormap based on the cumulative distribution of the loss. (B) The plot shows for different true models σ^{*2} the ratio of the local complexity measure by Jeffreys prior of the expected optimal to the true model.

The loss-minimizing value for σ^2 is given by the Bayesian mean which comes out as

$$\sigma^2(\hat\sigma^2_{\rm ML};\nu,\sigma_0^2) = \int {\rm d}\tilde\sigma^2 \tilde\sigma^2 \chi^{-2}(\tilde\sigma^2|\nu_N,\sigma_N^2) = \frac{\nu_N}{\nu_N-2}\sigma_N^2 = \frac{\nu\sigma_0^2 + \sum_i (x_i-\mu)^2}{\nu-2+N} = \frac{\frac{\nu-2}{N}\frac{\nu\sigma_0^2}{\nu-2} + \frac{1}{N}\sum_i(x_i-\mu)^2}{\frac{\nu-2}{N}+1} \tag{E9}$$

and we define

$$N_0 \equiv \nu - 2\,, \tag{E10}$$

$$\sigma^2_{\rm bias} \equiv \frac{\nu\sigma_0^2}{\nu-2}\,, \tag{E11}$$

$$\hat\sigma^2_{\rm ML} \equiv \frac{1}{N}\sum_i (x_i-\mu)^2\,. \tag{E12}$$

Up to a scaling of σ^{*2}/N, the empirical variance is distributed according to the chi-squared distribution of order N (recall, the variance is given by the sum of N i.i.d. squared Gaussian random variables divided by N) given by

$$\chi^2_N(x) = \frac{1}{2^{N/2}\Gamma(N/2)}\; x^{N/2-1}e^{-x/2}\,. \tag{E13}$$

Finally, we optimise the expected loss

$$\langle \mathcal{L}_{\sigma^*}(\sigma)\rangle_{\hat\sigma_{\rm ML}} = \int {\rm d}x\, \chi^2_N(x) D_{\rm KL}(P_{\sigma^{*2}}||P_{\sigma^2(\sigma^{*2}x/N;\nu,\sigma_0^2)}) \tag{E14}$$

with respect to the bias term, while κ is fixed. In this equation we are using the continuous version of the Kullback-Leibler divergence. By Eq. (18) of the main text, κ fixes q which in turn provides a value for N_0 given N:

$$q = \frac{N_0}{N} = \frac{\kappa}{1-\kappa}\,, \quad N_0 = Nq = \nu - 2\,, \tag{E15}$$

where we have also used the definition of N_0 in terms of the hyperparameter ν. For the hyperparameters ν and the bias we can thus write:

$$\nu = N_0 + 2\,, \tag{E16}$$

$$\sigma^2_{\rm bias} = \frac{N_0+2}{N_0}\sigma_0^2\,. \tag{E17}$$

Minimising the expected loss given N, yields the optimal choice for $\sigma^2_{\rm bias}$. In Figure S5 we show the optimal bias value as a function of the data size.

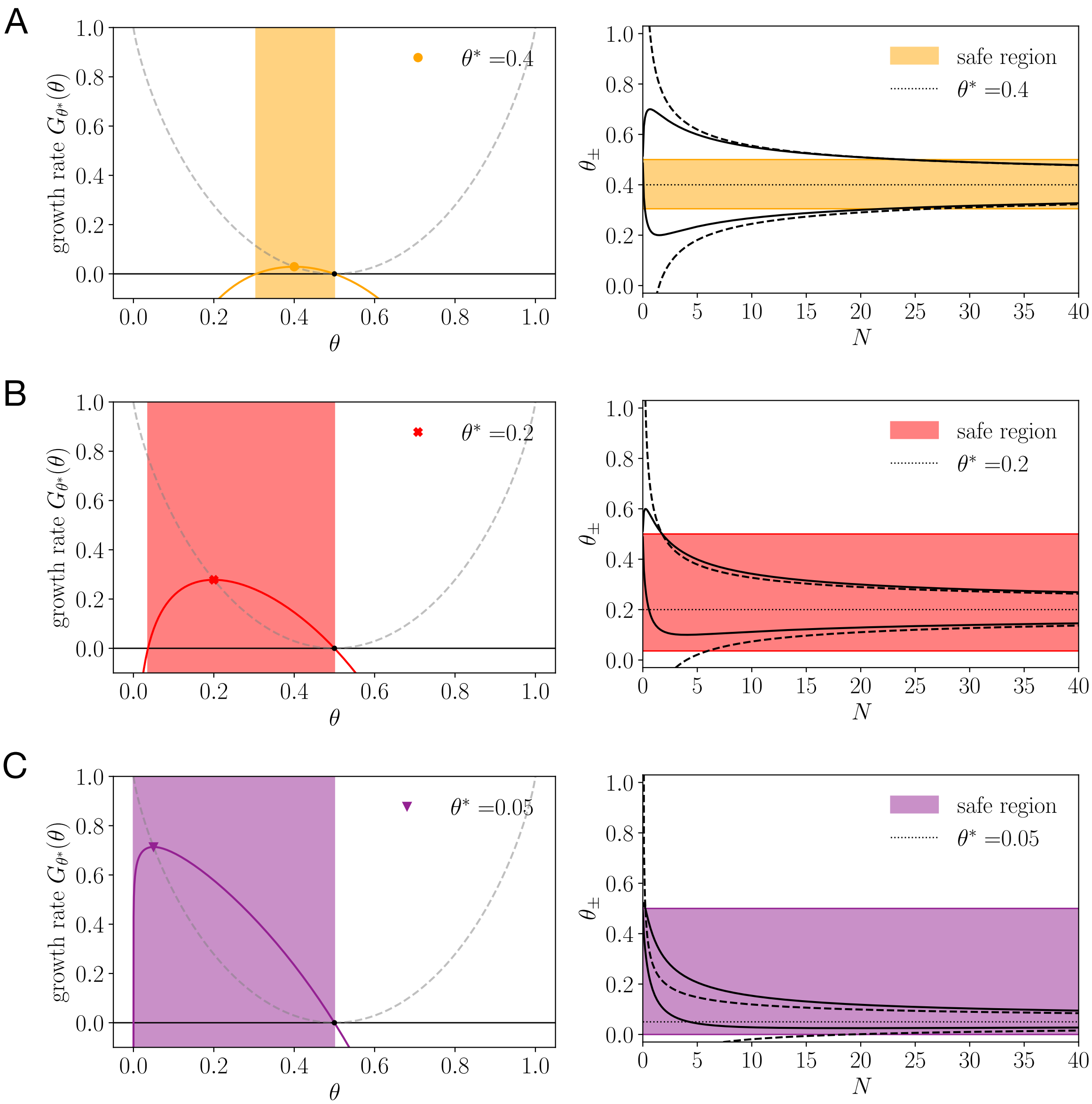

FIG. S6. The prior choice in Bayesian inference controls the safety of the learning strategy. Left column: long-term growth rate as a function of the adopted model ϕ for three different values of the true model $\theta^* = 0.4$, 0.2 and 0.05. Shaded boxes indicate the regions of non-negative growth rate. Right column: Magnitude of fluctuations of the conditional optimal model as a function of the number of observations N for two different learning strategies: maximum-likelihood estimation ($\alpha = 0$; dashed line) and Jeffreys prior ($\alpha = 1/2$; solid line).

Appendix F: Statistical fluctuations of different learning strategies

In Figure S6, we show the magnitude of statistical fluctuations of the inferred model in relation to the region of non-negative long-term growth rates. Subfigures A, B and C show different true models $\theta^* = 0.4$, 0.2 and 0.05, respectively. In the left column of subfigures we show the growth rates $G_{\theta^*}(\theta)$ as a function of the model choice θ. Regions where the growth rate is non-negative are indicated by color shaded boxes. In the right column of subfigures we show the statistical fluctuations of the inferred model as function of N for two different learning strategies. The first learning strategy is maximum-likelihood estimation $\alpha = 0$ and the second strategy uses $\alpha = 1/2$. The later strategy ($\alpha = 1/2$) is safer, because the magnitude of fluctuations is shrunk and more contained in the interval of non-negative growth rates in comparison to the maximum-likelihood strategy.
